# From Vaccine Skepticism to Institutional Distrust: The Post-Pandemic Shift

**DOI:** 10.3390/vaccines14070622

**Published:** 2026-07-16

**Authors:** Francesco De Maria, Francesco Branda, Giancarlo Ceccarelli, Fabio Scarpa, Massimo Ciccozzi, Alessandro Russo

**Affiliations:** 1 Infectious and Tropical Diseases Unit, “Renato Dulbecco” Teaching Hospital of Catanzaro, 88100 Catanzaro, Italy; francescodemaria16@gmail.com (F.D.M.); a.russo@unicz.it (A.R.); 2 Unit of Medical Statistics and Molecular Epidemiology, Università Campus Bio-Medico di Roma, 00128 Rome, Italy; m.ciccozzi@unicampus.it; 3 Department of Public Health and Infectious Diseases, University Hospital Policlinico Umberto I, Sapienza University of Rome, 00185 Rome, Italy; giancarlo.ceccarelli@uniroma1.it; 4 Department of Biomedical Sciences, University of Sassari, 07100 Sassari, Italy; fscarpa@uniss.it

**Keywords:** vaccine hesitancy, institutional trust, institutional hesitancy, COVID-19 pandemic, public health, health communication, misinformation, trust in science, vaccination policy

## Abstract

**Background**: Vaccine hesitancy has traditionally been understood as a multifactorial phenomenon shaped by individual beliefs, risk perceptions, and access barriers. However, the COVID-19 pandemic has fundamentally transformed the relationship between citizens, science, and public institutions, raising the question of whether vaccine hesitancy has evolved into a broader form of institutional distrust. **Objective**: This narrative review synthesizes evidence on vaccine confidence, trust dynamics, misinformation, and post-pandemic attitudes to propose a new conceptual framework, i.e., institutional hesitancy, that reframes vaccine acceptance within the wider context of institutional credibility, transparency, and legitimacy. **Methods**: We conducted a narrative synthesis of peer-reviewed literature, surveillance reports, and cross-national surveys published between 2015 and 2026, focusing on trust in science, governments, public health agencies, healthcare systems, and regulatory authorities as determinants of vaccination behavior. **Results**: The evidence consistently demonstrates that institutional trust is among the strongest predictors of vaccine acceptance, often surpassing traditional demographic and knowledge-based variables. The pandemic exposed and amplified pre-existing fractures in the relationship between citizens and institutions, creating a legacy of institutional skepticism that extends beyond COVID-19 vaccines to routine immunization programs, seasonal vaccination campaigns, and future pandemic preparedness. Traditional information-based approaches, which assume that knowledge deficits drive hesitancy, have proven insufficient when trust is compromised. Instead, rebuilding vaccine confidence requires sustained investment in institutional transparency, community engagement, and accountable governance. **Conclusions**: The post-pandemic era calls for a fundamental reconceptualization of vaccine hesitancy. We propose the institutional hesitancy framework as a complementary lens that shifts the analytical focus from individual knowledge deficits to relational dynamics between citizens and institutions. Addressing this challenge requires moving beyond communication campaigns toward long-term strategies that restore institutional trust and strengthen the resilience of public health systems.

## 1. Introduction

Vaccination remains one of the most successful public health interventions in human history, preventing millions of deaths annually and contributing substantially to gains in life expectancy worldwide. From the eradication of smallpox to the dramatic reduction in morbidity and mortality from vaccine-preventable diseases such as measles, polio, and diphtheria, immunization programs have fundamentally transformed global health over the past century. Despite this proven effectiveness, vaccine acceptance has never been universal. Concerns regarding vaccine safety, effectiveness, necessity, and trustworthiness have accompanied immunization programs for decades, giving rise to what is commonly referred to as vaccine hesitancy [1,2].

Long before the emergence of COVID-19, vaccine hesitancy had been recognized as a growing global challenge. In 2014, the Strategic Advisory Group of Experts (SAGE) on Immunization established a formal definition of vaccine hesitancy and proposed the influential “3C” model, identifying confidence, complacency, and convenience as the core domains shaping vaccination decisions [3]. Building on this framework, in 2019 the World Health Organization identified vaccine hesitancy as one of the top ten threats to global health, reflecting growing concerns that declining confidence in vaccination could undermine achievements in disease prevention and control [2,4]. Traditionally, vaccine hesitancy has been conceptualized as a multifactorial phenomenon influenced by individual beliefs, perceptions of risk, cultural values, access barriers, and confidence in vaccines and healthcare systems [2,4,5]. The widely adopted “3C” model, identifying confidence, complacency, and convenience as the three core domains shaping vaccination decisions, has provided a useful framework for understanding the psychological and structural determinants of hesitancy [3,6]. Subsequent refinements, including the “5C” model (which adds calculation and collective responsibility), have further enriched this conceptual landscape [7].

The COVID-19 pandemic dramatically transformed this landscape. Never before had vaccines been developed, authorized, distributed, and discussed under such intense public scrutiny. The unprecedented speed of vaccine development, enabled by decades of prior research on mRNA technology, generated both admiration and suspicion. Scientific debates that would normally occur within academic circles became highly visible to the general public, with experts disputing recommendations on masking, booster doses, vaccine schedules, and public health restrictions in real time. Public health recommendations evolved as evidence accumulated, a normal feature of the scientific process, but one that many interpreted as inconsistency, incompetence, or evidence of hidden agendas. Governments introduced unprecedented containment measures, including lockdowns, travel restrictions, and vaccine mandates, while social media platforms amplified both accurate information and misinformation at an unprecedented scale [8,9,10,11,12]. The resulting “infodemic” exposed populations to a relentless stream of conflicting narratives, conspiracy theories, and emotionally charged content that often overwhelmed efforts at evidence-based communication.

As a result, vaccine acceptance became deeply intertwined with broader societal issues, including political polarization, institutional credibility, media trust, and perceptions of scientific authority [13,14,15,16]. Attitudes toward COVID-19 vaccines increasingly reflected pre-existing political identities, with vaccination status becoming a marker of ideological affiliation in many countries. Public health interventions that would have been uncontroversial in previous decades became flashpoints in broader cultural and political conflicts. Although much of the existing literature continues to frame vaccine hesitancy primarily as an individual-level behavioral problem, one that can be addressed through better education, improved risk communication, or enhanced access to vaccination services, emerging evidence suggests that the phenomenon may have evolved into something more complex and structurally embedded [17,18,19].

In many settings, reluctance toward vaccination appears to reflect not only concerns about vaccines themselves but also declining confidence in the institutions responsible for developing, regulating, recommending, and communicating health interventions [17,18,19,20,21,22,23,24,25]. Surveys conducted across different countries have consistently shown that trust in governments, public health authorities, healthcare systems, pharmaceutical companies, and scientific institutions is among the strongest predictors of vaccine acceptance [18,19,22,26,27,28,29,30]. Individuals who express low trust in these institutions are significantly less likely to accept vaccination, regardless of their level of education, health literacy, or access to accurate information. This pattern has been observed across diverse populations and cultural contexts, suggesting that the relationship between institutional trust and vaccine acceptance is not a localized phenomenon but a global trend with profound implications for public health.

This observation raises an important question: has the COVID-19 pandemic transformed vaccine hesitancy into a broader form of institutional hesitancy? The present narrative review explores this possibility. We argue that the most enduring legacy of the pandemic may not be vaccine hesitancy itself, but rather a profound reconfiguration of the relationship between citizens, science, and public institutions. By examining evidence on vaccine confidence, misinformation, trust dynamics, risk communication, and post-pandemic attitudes toward public health authorities, we propose that vaccine acceptance should increasingly be understood within a wider framework of institutional trust, one that acknowledges the central role of credibility, transparency, and legitimacy in shaping health behaviors. This perspective has important implications not only for understanding the dynamics of the COVID-19 pandemic but also for designing effective vaccination strategies for routine immunization programs and future pandemic preparedness.

We conducted a systematic literature search of PubMed/MEDLINE, Scopus, and Web of Science for peer-reviewed articles published between January 2015 and June 2026, supplemented by Google Scholar and official sources (World Health Organization (WHO), European Centre for Disease Prevention and Control (ECDC), Centers for Disease Control and Prevention (CDC), Kaiser Family Foundation (KFF)) for grey literature, surveillance reports, and tracking poll data not indexed in bibliographic databases. Search terms combined “vaccine hesitancy”, “vaccine confidence”, “vaccine refusal”, “institutional trust”, “trust in science”, “trust in government”, “COVID-19”, “misinformation”, and “public health communication”, using Boolean operators and truncation. We included empirical (survey, cross-national, longitudinal) and theoretical/conceptual articles addressing determinants of vaccine attitudes with explicit attention to trust or institutional variables; we excluded case reports, non-English/non-Italian sources without an available translation, and opinion pieces lacking original data or a stated conceptual argument. Consistent with the narrative, rather than systematic, design of this review, study identification and selection were purposive and iterative, guided by thematic saturation rather than a pre-registered protocol.

The review is structured as follows. Section 2 traces the transformation of vaccine hesitancy, beginning with an examination of pre-pandemic frameworks and their limitations, followed by an analysis of how the COVID-19 pandemic eroded institutional trust across multiple dimensions, and culminating in the proposal of the institutional hesitancy framework as a new conceptual lens. Section 3 reviews the empirical evidence supporting this framework and critically evaluates the limitations of traditional information-based approaches to addressing vaccine hesitancy. Section 4 discusses the implications of institutional hesitancy for vaccination programs and outlines strategies for restoring institutional trust in the post-pandemic era. Section 5 provides a critical discussion of the findings, acknowledges the limitations of the review, and outlines priorities for future research. Section 6 concludes with the key messages and their implications for public health policy and practice.

## 2. The Transformation of Vaccine Hesitancy: Before, During, and After COVID-19

### 2.1. Pre-Pandemic Vaccine Hesitancy

Before the COVID-19 pandemic, vaccine hesitancy was generally understood as a spectrum of attitudes ranging from uncertainty to delayed acceptance and outright refusal despite vaccine availability [1,2,31]. Importantly, hesitancy was never considered synonymous with anti-vaccination beliefs. Whereas anti-vaccination activism typically involves active opposition to vaccination based on deeply held ideological convictions, hesitancy encompasses a broader and more nuanced range of attitudes, including passive reluctance, ambivalence, situational delays, and varying degrees of acceptance that may change over time or across different vaccines. It represented a complex and context-specific phenomenon shaped by psychological, social, cultural, and structural determinants, varying not only between individuals but also across time, geographic settings, and specific vaccines [2,31,32]. This contextual variability was a key insight of early research; an individual who hesitates about one vaccine may readily accept another, and hesitancy in one setting may not predict hesitancy in another.

The Strategic Advisory Group of Experts (SAGE) on Immunization developed the influential “3C” framework, identifying *confidence* (trust in vaccine safety, effectiveness, and the institutions that recommend them), *complacency* (low perceived disease risk), and *convenience* (practical access barriers) as the three core determinants of hesitancy [3,6]. This was later expanded into the “5C” model, which added *calculation* (deliberate risk-benefit weighing) and *collective responsibility* (motivation to protect others) [7]. In 2019, the World Health Organization elevated vaccine hesitancy to one of the top-ten global health threats, acknowledging its potential to undermine decades of progress [4]. While trust was central to these frameworks, it was generally considered one determinant among several, alongside knowledge, risk perception, and access, rather than the primary lens through which other factors were interpreted.

Before COVID-19, studies consistently identified concerns regarding vaccine safety, fear of adverse events, low disease risk perception, limited health literacy, and exposure to misinformation as major determinants of hesitancy [31,32,33,34,35,36]. Concerns about vaccine safety were particularly prominent, often fueled by historical controversies such as the now-debunked link between the MMR vaccine and autism, which continued to influence public perceptions long after the scientific consensus had been firmly established and the original study retracted. Fear of adverse events, including both common side effects and extremely rare but serious complications, also played a significant role in shaping individual attitudes, with some individuals overestimating the probability of rare adverse events while underestimating the risks of vaccine-preventable diseases. Limited health literacy, defined as the capacity to obtain, process, and understand basic health information needed to make appropriate health decisions, was consistently associated with lower vaccine acceptance, as individuals with lower health literacy often struggled to evaluate complex risk-benefit information. Importantly, trust already occupied a central position within these models. However, trust was generally considered one determinant among many rather than the dominant explanatory factor [2,35,37]. Within the 3C framework, confidence represented one of three equally weighted domains, rather than the primary lens through which other determinants were interpreted. This conceptual positioning reflected a broader assumption in public health that trust was just one of several factors influencing vaccination decisions, alongside access, risk perception, and knowledge.

The concept of vaccine confidence further emphasized this dimension. Larson and colleagues, in a landmark 67-country survey published in 2016, demonstrated substantial global variability in confidence toward vaccines, highlighting that acceptance was strongly influenced by trust in health systems and scientific authorities [2]. Countries with high levels of trust in healthcare professionals and government health agencies consistently exhibited higher vaccine confidence, whereas regions with historical experiences of medical marginalization, institutional distrust, or recent vaccine safety controversies showed substantially lower confidence levels. The study also revealed that vaccine confidence was not static but varied significantly across countries and over time, influenced by local events, media coverage, and political contexts. Later work reinforced the idea that vaccine confidence is dynamic, fragile, and susceptible to rapid fluctuations in response to social and political events [38]. Confidence can be eroded by vaccine safety scares, political controversies, perceived failures in public health communication, or high-profile adverse events, and it can be rebuilt through transparent communication, community engagement, and consistent institutional behavior over time. This volatility would become particularly evident during the COVID-19 pandemic, when confidence in vaccines fluctuated dramatically in response to rapidly evolving circumstances.

Nevertheless, most pre-pandemic frameworks retained a relatively individual-centered perspective. Hesitancy was commonly viewed as a problem arising from insufficient knowledge, cognitive biases, risk perceptions, or personal beliefs. The underlying assumption was that individuals who declined or delayed vaccination did so primarily because they lacked accurate information or held irrational beliefs that could be corrected through education. This “deficit model” of public understanding of science, the assumption that public resistance to scientific recommendations stems from knowledge deficits that can be remedied through better communication, dominated public health thinking for decades. Solutions therefore focused primarily on educational interventions, communication campaigns, and improving access to vaccination services [3,6,39]. Public health authorities invested heavily in developing informational materials, fact sheets, and public service announcements designed to correct misconceptions and provide clear, evidence-based guidance. Healthcare professionals were encouraged to engage in motivational interviewing, address patient concerns, and share persuasive vaccine information during clinical encounters. These approaches assumed that more information and better access would naturally translate into higher uptake, an assumption that, as the COVID-19 pandemic would starkly demonstrate, may have been overly optimistic. The persistence of hesitancy despite unprecedented access to scientific information during the pandemic exposed the limitations of the deficit model and suggested that trust, far from being merely one determinant among many, had become the central axis around which vaccination decisions increasingly revolve. This realization would challenge the foundational assumptions of pre-pandemic vaccination programs and set the stage for a fundamental rethinking of how vaccine hesitancy is understood and addressed.

### 2.2. The Pandemic’s Impact on Institutional Trust

Although vaccine hesitancy has often been framed as a problem of misinformation, risk perception, or inadequate health literacy, an increasing body of evidence suggests that trust may represent the single most important determinant of vaccine acceptance in the post-pandemic era [17,18,26,27,37,40]. Indeed, the COVID-19 crisis exposed and, in many cases, dramatically amplified pre-existing fractures in the relationship between citizens and institutions. Trust, which had been eroding gradually in several countries over previous decades, experienced accelerated decline during the pandemic, with profound consequences for public health behavior and institutional legitimacy. The pandemic did not create these fractures from nothing; rather, it revealed and deepened vulnerabilities that had been developing for years, driven by growing political polarization, economic inequality, historical injustices, and the erosion of shared social narratives.

Trust is a multidimensional construct encompassing confidence in scientific expertise, healthcare professionals, public health agencies, regulatory authorities, governments, and pharmaceutical companies [21,23,37]. Each of these dimensions plays a distinct but interrelated role in shaping vaccine attitudes, and each was subjected to extraordinary public scrutiny during the pandemic. Unlike previous health crises, where trust could be taken relatively for granted, the COVID-19 pandemic placed institutional credibility under a microscope, exposing tensions and contradictions that had previously remained hidden from public view. The cumulative effect was a broad-based erosion of confidence that extended well beyond vaccines themselves, affecting perceptions of science, governance, and the very nature of expert authority.

One of the most significant shifts involved trust in science itself. Traditionally, scientific institutions have benefited from relatively high levels of public credibility across most societies. The authority of science rested on a combination of demonstrated effectiveness, professional norms of objectivity, and the perception that scientific knowledge is produced through rigorous, disinterested methods. However, the visibility of scientific disagreements, changing recommendations, and the politicization of scientific findings contributed to a perception among some groups that scientific knowledge was uncertain, inconsistent, or influenced by external interests [24,41]. Scientists, who had previously been viewed as neutral experts, were increasingly perceived by some as partisan actors embedded within broader political and institutional networks. The very mechanisms that make science reliable in the long term, including peer review, replication, and the willingness to revise conclusions in light of new evidence, became, in the short term, sources of public confusion and suspicion. Several studies conducted during the pandemic documented measurable declines in public trust in science, with the decline most pronounced among individuals who identified with political groups that had been critical of pandemic responses [24]. Importantly, studies conducted in multiple countries have demonstrated a strong and consistent association between trust in science and vaccine acceptance, with individuals reporting higher confidence in scientific institutions being significantly more likely to receive COVID-19 vaccines [18,26,27]. Conversely, those who expressed low trust in science were more likely to delay or refuse vaccination, regardless of their level of formal education or access to accurate information.

At the same time, confidence in governments emerged as a critical predictor of vaccine uptake, perhaps even more influential than trust in science alone. Several investigations found that distrust toward governmental institutions was independently associated with vaccine hesitancy, regardless of educational level, health literacy, or perceived disease risk [19,29,42]. Individuals who viewed their governments as untrustworthy, incompetent, self-interested, or opaque were significantly more likely to express reluctance toward COVID-19 vaccination. This pattern was observed across diverse political and cultural contexts, suggesting that the relationship between political trust and vaccine acceptance is not confined to any particular country or political system. In some settings, vaccination became a symbolic expression of political identity, transforming a public health intervention into a marker of support for, or opposition to, governmental authority [13,16]. The politicization of vaccination was particularly evident in countries with deep pre-existing political divisions, where attitudes toward COVID-19 vaccines aligned closely with party identification and where vaccine acceptance or refusal became a way of expressing broader ideological commitments.

The United States illustrates a further complication; institutional trust and vaccine attitudes do not always align in the intuitive direction. Vaccine hesitancy has been disproportionately concentrated among voters who report relatively high trust in the political leadership in power, even as that same leadership has invested in pharmaceutical development and taken credit for the speed of vaccine delivery. This apparent paradox is more consistent with the institutional hesitancy framework than it first appears once distinct institutional targets are distinguished: technical and scientific agencies (e.g., the CDC and NIH) can be selectively targeted for blame, for example for revising guidance as evidence evolved, a normal feature of the scientific process, while trust in political leadership itself remains comparatively high. In this configuration, institutional distrust is not diffuse but is directed, and can be actively cultivated for strategic political advantage, turning a routine feature of scientific self-correction into evidence of institutional incompetence or bad faith rather than an opportunity to explain how science operates. This dynamic underscores that shifts in institutional trust are not purely emergent societal drift but can be a contested and, at times, deliberately shaped resource. Consistent with this, subsequent changes in the composition of federal health agencies, including the replacement of career scientific staff with political appointees, raise the possibility that the direction of this relationship may invert over time, with constituencies that previously trusted these agencies becoming skeptical, and vice versa; existing empirical studies, collected predominantly between 2020 and 2023, do not yet capture this potential reversal, which we identify as a priority for future longitudinal research.

Relatedly, recent tracking poll data (KFF Health Information and Trust survey) indicate that having a trusted primary care provider is associated with lower uptake of common vaccine-related misinformation, reinforcing the point developed further in Section 4 that trust operating at the local, proximal level of an individual provider can matter more for behavior than trust in distal national institutions, even when that provider is embedded within, and dependent on, institutions the individual does not otherwise trust.

This transformation of a public health measure into a political statement was unprecedented in modern history and had profound implications for both vaccine uptake and social cohesion.

Public health agencies also experienced substantial changes in public confidence during and after the pandemic. While many individuals relied heavily on national and international health authorities during the early phases of the pandemic for guidance on risk mitigation, testing, and vaccination, prolonged emergency measures, evolving recommendations, and perceived communication failures contributed to declining confidence among certain segments of the population [17,18,19]. Longitudinal data from Canada, collected at multiple time points before, during, and after the pandemic, documented measurable changes in trust toward public health institutions [19]. Similarly, the multinational study by Lazarus and colleagues across 23 countries found that confidence in health information sources and public institutions had become more variable and fragile by 2023 compared to pre-pandemic baselines [18]. Several factors contributed to this decline: the sheer duration of the pandemic led to fatigue and skepticism; the inconsistency of recommendations across different jurisdictions created confusion; high-profile communication missteps were seized upon by critics as evidence of incompetence or dishonesty; and the unprecedented visibility of internal scientific debates eroded the perception that public health authorities spoke with a unified voice. The cumulative effect was an erosion of the credibility that public health agencies had traditionally enjoyed.

The pharmaceutical sector represented another important dimension of public trust. The unprecedented speed of vaccine development, achieved through years of prior research on mRNA technology, massive public investment, and streamlined regulatory processes, generated both admiration and suspicion. For many individuals, the rapid development of effective vaccines was a triumph of scientific innovation and collaboration. For others, however, the speed of development raised concerns about safety, corners being cut, and commercial interests overriding public health priorities [22,23]. The involvement of large pharmaceutical companies, which had historically been viewed with skepticism by some segments of the public, further complicated attitudes. Recent evidence suggests that confidence in manufacturers and regulatory authorities remains a significant predictor of acceptance not only for COVID-19 vaccines but also for newer vaccine technologies such as mRNA platforms [22]. Individuals who expressed trust in the regulatory process, and who believed that vaccines had undergone rigorous safety evaluation despite the accelerated timeline, were more likely to accept vaccination. Conversely, those who perceived regulatory authorities as captured by commercial interests or as insufficiently transparent were more likely to express hesitancy.

Healthcare professionals, particularly physicians and nurses, have traditionally enjoyed high levels of public trust, and this trust largely persisted through the pandemic. Surveys conducted across multiple countries consistently place healthcare workers among the most trusted sources of health information, often ranking above government officials, public health agencies, and media outlets [41,43]. Despite immense strain, burnout, and staffing shortages, frontline healthcare workers remained among the most trusted advisors for vaccination decisions in most settings. Nevertheless, trust in healthcare systems as institutions, rather than individual healthcare workers, faced significant challenges. Overwhelmed hospitals, delayed elective procedures, and inequitable access to care created perceptions that the healthcare system was failing to meet community needs. For historically marginalized populations, experiences of discrimination in healthcare settings have shaped vaccine attitudes in ways that extend beyond vaccine-specific concerns [30,40].

Importantly, trust is not distributed equally across societies. Historical experiences, cultural context, social inequalities, discrimination, and previous interactions with healthcare systems can profoundly influence institutional credibility [30,40,42,44]. In some populations, particularly historically marginalized groups, vaccine hesitancy may reflect broader experiences of exclusion and institutional distrust rather than concerns specific to vaccination itself. Understanding these historical and structural dimensions of trust is essential for designing effective, equitable vaccination strategies. Furthermore, recent research suggests that trust functions as a mediating variable that shapes how other determinants, such as misinformation, educational messages, or risk communication, influence vaccine attitudes. Exposure to misinformation alone does not uniformly lead to vaccine refusal; its effects appear to be mediated by trust in institutions and scientific authorities [28,45,46]. Individuals with high institutional trust are more likely to reject misinformation when they encounter it, whereas those with low institutional trust are more susceptible to its influence. Likewise, educational attainment does not consistently predict vaccine acceptance when institutional distrust is high; highly educated individuals who distrust institutions may be just as likely to refuse vaccination as those with lower educational levels [29,42]. These observations suggest that trust functions not merely as one determinant among many but as a central lens through which all other determinants are interpreted.

Collectively, these findings suggest that the central challenge facing vaccination programs may no longer be limited to vaccine confidence. Instead, vaccine attitudes increasingly appear to be embedded within a wider ecosystem of institutional trust, one in which confidence in vaccines depends on confidence in the institutions responsible for their development, regulation, recommendation, and communication. This shift has profound implications for public health. Trust is not easily rebuilt once it has been eroded. Unlike knowledge deficits, which can be addressed through education, trust requires sustained effort, demonstrated competence, transparency, and accountability over time. During a crisis, trust may be rapidly lost but only slowly regained. The pandemic has therefore created a legacy of institutional skepticism that may persist for years or even decades, affecting not only COVID-19 vaccination but also routine immunization programs, acceptance of future vaccines, and compliance with public health measures during future emergencies. The emergence of this trust deficit sets the stage for the conceptual framework introduced in the next section: institutional hesitancy.

The evidence accumulated since the COVID-19 pandemic invites a fundamental reconsideration of how vaccine hesitancy is conceptualized. Existing frameworks, including the influential “3C” and “5C” models, remain valuable for understanding individual-level determinants of vaccination behavior, providing structured approaches to assessing confidence, complacency, convenience, calculation, and collective responsibility [3,5,6,7]. These models have proven useful in guiding research, informing interventions, and shaping public health communication strategies. However, they may be insufficient to explain the broader societal dynamics that have emerged over the last several years, dynamics in which vaccine attitudes appear increasingly shaped by perceptions of institutional legitimacy, political polarization, and the erosion of social trust. The pandemic has revealed that vaccine hesitancy is not merely an individual behavioral problem but a phenomenon deeply embedded within wider social, political, and institutional contexts.

Institutional hesitancy is not proposed as a wholly new determinant standing alongside those already identified by classical frameworks, but as a reweighting of them: whereas the 3C and 5C models treat confidence as one coordinate factor among several (complacency, convenience, constraints, calculation, collective responsibility), institutional hesitancy proposes that trust in institutions functions as a superordinate filter that conditions how all of the other determinants, including knowledge, risk perception, and exposure to misinformation, are processed and weighted by the individual. It is similarly distinct from “vaccine confidence”, which is an outcome-level, vaccine-specific attitude, and from diffuse “general institutional trust”, a civic disposition that is not necessarily activated in health contexts; institutional hesitancy is best understood as the mechanism that links the latter to the former.

The framework can also be situated relative to established behavioral theories. In the Health Belief Model, perceived barriers and external cues to action are frequently institution-mediated, for example, the accessibility and perceived legitimacy of a vaccination program shape the practical and psychological barriers an individual perceives. In the Theory of Planned Behavior, subjective norms and perceived behavioral control are shaped by confidence in the institutions that structure vaccine recommendation, endorsement, and access. Kopasz et al. [26] provide an empirical bridge for this integration, applying an extended Theory of Planned Behavior model in which trust in science operates on vaccination intention indirectly, through perceived risk. Institutional hesitancy is therefore proposed as a higher-order construct that can be nested within, rather than substituted for, HBM- or TPB-based models of vaccination behavior.

We propose that many contemporary manifestations of vaccine hesitancy can be understood within a broader framework that may be described as institutional hesitancy. This concept captures a dimension of vaccine reluctance that extends beyond concerns about the vaccine itself to encompass skepticism, uncertainty, or distrust toward the institutions responsible for producing, regulating, recommending, and communicating health interventions. Institutional hesitancy can be defined as a state of skepticism, uncertainty, or distrust toward the institutions responsible for producing, regulating, recommending, and communicating health interventions. Under this framework, the vaccine itself is not necessarily the primary object of concern. Rather, vaccination becomes a visible expression of deeper attitudes toward scientific authorities, healthcare systems, governments, regulatory agencies, and other institutions involved in public health decision-making. An individual who declines vaccination may be expressing, consciously or unconsciously, a broader skepticism about the credibility of the institutions that recommend it, rather than a specific concern about the vaccine’s safety or effectiveness.

**Figure 1:**
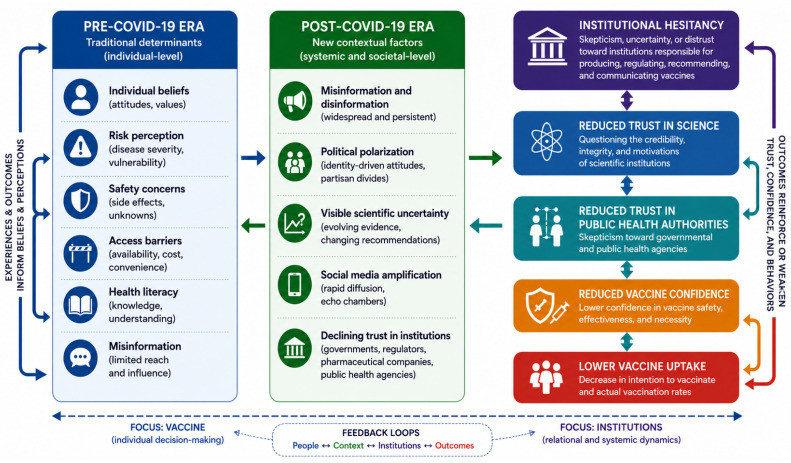
Conceptual framework illustrating the proposed shift from traditional vaccine hesitancy to institutional hesitancy in the post-COVID-19 era. The left side depicts classical vaccine hesitancy, primarily driven by individual-level determinants such as confidence, complacency, convenience, and risk perception. The right side represents institutional hesitancy, in which broader societal and institutional factors, including misinformation, political polarization, scientific uncertainty, and declining trust in institutions, shape vaccine confidence and uptake. The model highlights the transition from a vaccine-centered perspective to a trust-centered interpretation of vaccination behavior, while incorporating feedback loops that capture the dynamic, bidirectional nature of these relationships: contextual factors are both influenced by and influence institutional behavior and public responses, creating reinforcing cycles that can stabilize or erode vaccine confidence over time.

The conceptual transition from traditional vaccine hesitancy to this broader framework of institutional hesitancy is illustrated in Figure 1. The figure depicts the shift from a vaccine-centered perspective, in which hesitancy is primarily driven by individual-level determinants such as confidence, complacency, and convenience, to a trust-centered interpretation of vaccination behavior. Crucially, the figure also incorporates feedback loops that capture the dynamic, bidirectional nature of these relationships: contextual factors, including misinformation, political polarization, and visible scientific uncertainty, do not merely influence individual attitudes in a unidirectional manner; rather, they are themselves shaped and reinforced by institutional behavior and public responses. Individuals’ experiences with institutions, in turn, feedback into the broader social and political context, creating reinforcing cycles that can either stabilize or erode vaccine confidence over time. In this new framework, broader societal and institutional factors, including misinformation, political polarization, scientific uncertainty, and declining trust in institutions, shape vaccine confidence and uptake. The model highlights that vaccine attitudes are increasingly embedded within a wider ecosystem of institutional trust, where confidence in vaccines depends on confidence in the institutions responsible for their development, regulation, and recommendation.

This distinction is important because it shifts the analytical focus from individual knowledge deficits to relational dynamics between institutions and citizens. In traditional models, vaccine refusal is often interpreted as the consequence of misinformation, inadequate education, or cognitive biases. The underlying assumption is that if individuals were simply better informed, they would make the rational choice to accept vaccination. While these factors undoubtedly contribute to vaccine attitudes, they do not fully explain why similar information may be interpreted differently by individuals with differing levels of institutional trust [26,28,45]. Two individuals with identical access to scientific information may reach opposite conclusions about vaccination depending on their prior trust in the institutions providing that information. This observation suggests that trust is not merely one determinant among many but a fundamental lens through which all other determinants are filtered. The same message that is persuasive when delivered by a trusted source may be dismissed or rejected when delivered by an untrusted one, regardless of its factual content.

To clarify the distinction between traditional vaccine hesitancy and the broader concept of institutional hesitancy proposed in this review, several key dimensions merit comparison. In classical vaccine hesitancy, the primary object of concern is the vaccine itself, including its safety, effectiveness, necessity, and potential side effects [1,2,5,6,7,31,32]. The analytical focus is on individual attitudes and decision-making processes, with main determinants including confidence, complacency, convenience, constraints, calculation, and collective responsibility [3,5,6,7]. Trust, within this framework, is one determinant among several influencing vaccine acceptance [2,35,37]. Misinformation is understood to directly influence vaccine attitudes and risk perceptions [9,10,11,12,31,32,33,34,35,36], while scientific uncertainty is viewed primarily as a challenge for risk communication [14,39]. The primary explanation for refusal or delay is concern about vaccine safety, efficacy, or adverse events [31,32,33,34,35,36], and the communication model emphasizes information provision and correction of misconceptions [3,6,39]. Public health interventions focus on education campaigns, improved access, and vaccine promotion [3,5,6,7,39], with the underlying question being “Do I trust this vaccine?” The expected outcome of successful intervention is increased vaccine confidence and uptake [3,5,6,7,31,32].

In contrast, institutional hesitancy shifts the primary object of concern to the institutions responsible for developing, regulating, recommending, and communicating vaccines [17,18,19,20,21,22,23,24,25]. The analytical focus becomes the relationship between citizens and institutions [17,18,19,23,25], with main determinants including trust in science, governments, public health agencies, healthcare systems, and regulatory authorities [18,19,22,26,27,28,29,30]. Trust occupies a central position as the framework through which information and recommendations are interpreted [17,18,19,26,27,28,29,30]. Misinformation is understood to amplify pre-existing distrust toward institutions and experts rather than directly causing vaccine refusal [28,45,46,47,48]. Scientific uncertainty may be perceived as evidence of institutional inconsistency or lack of credibility [14,24,41]. The primary explanation for refusal or delay is skepticism regarding the legitimacy, transparency, or trustworthiness of institutions [17,20,23,25]. The communication model emphasizes dialogue, transparency, community engagement, and trust-building [49,50,51,52,53], while public health interventions focus on institutional transparency, stakeholder engagement, accountability, and long-term trust restoration [17,21,50,51,52,53]. The underlying question becomes “Do I trust the institutions behind this vaccine?” and the expected outcome is restoration of institutional trust, leading to sustainable vaccine confidence and broader public health resilience [17,18,19,20,21,50,51,52,53]. This conceptual comparison is summarized in Table 1, which highlights the fundamental shift in perspective that the institutional hesitancy framework represents.

**Figure 2:**
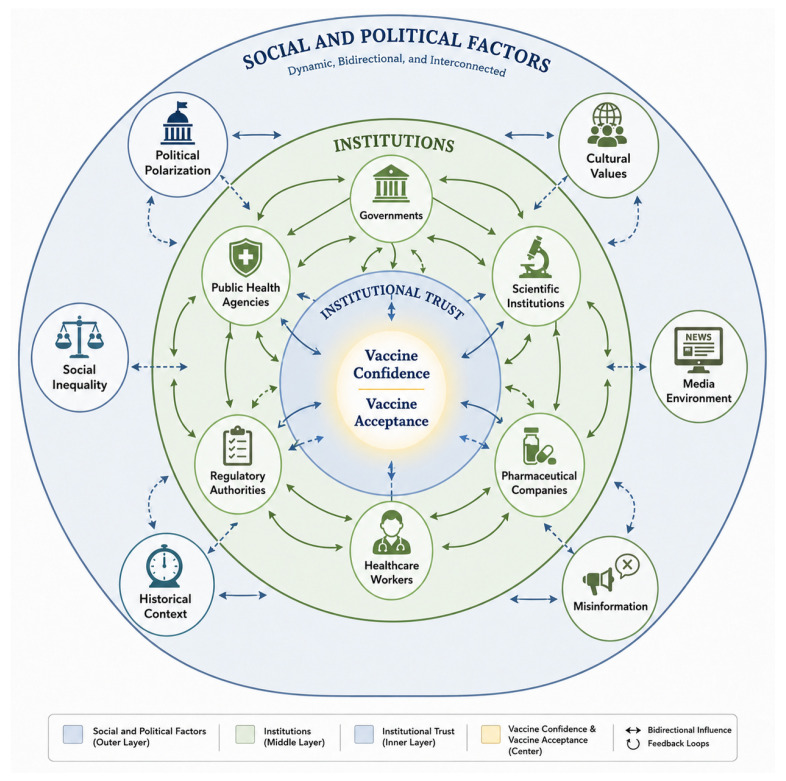
Conceptual diagram illustrating the multi-layered ecosystem of trust influencing vaccination decisions. The outermost ring captures the broader social and political context, including political polarization, social inequality, historical experiences, cultural values, and the media environment. The middle ring encompasses the key institutional actors involved in vaccine development and delivery: governments, public health agencies, scientific institutions, regulatory authorities, pharmaceutical companies, and healthcare workers. The inner ring represents institutional trust itself, while the center represents vaccine confidence and acceptance. Unlike a unidirectional model, the arrows depict bidirectional influence and feedback loops: contextual factors shape institutional credibility and behavior, while institutional actions, through communication, transparency, and responsiveness, feed back into the broader social and political environment. Similarly, institutional trust is not merely a precursor to vaccine confidence but is itself shaped by institutional conduct and public responses, creating iterative cycles that can reinforce or erode trust over time.

The complex, multi-layered nature of institutional trust and its influence on vaccination decisions is further illustrated in Figure 2. Importantly, the figure depicts trust not as a static hierarchy but as a dynamic, bidirectional ecosystem: the outermost layer (social and political context) shapes institutional behavior and credibility, while institutional actions, in turn, feed back into the broader social and political environment through public perceptions, media coverage, and political discourse. Similarly, trust in institutions is not merely a precursor to vaccine confidence but is itself shaped by institutional behavior, communication, and responsiveness, creating iterative feedback cycles that can either reinforce or undermine trust over time. An important refinement to this trust ecosystem is that its layers are only loosely coupled: trust in national government, in federal or national health agencies, and in local providers or community organizations can diverge substantially, and vaccination decisions appear most proximally shaped by trust in whichever node in this chain is closest to the individual, often a local pediatrician, nurse, or community health worker, rather than by trust in the most distal national institutions. This is consistent with reports from clinicians engaged in vaccine confidence work that parents will bring children in for vaccination on the strength of trust in “someone you trust” locally, even amid broadly declining national institutional trust.

This dynamic can be leveraged strategically. In the United States, the Office of Minority Health’s National COVID-19 Resiliency Network, led by Morehouse School of Medicine, illustrates how a federal institution can rebuild trust indirectly: rather than communicating directly with hesitant populations, the initiative funded and empowered national and community-based organizations to develop and deliver culturally and linguistically tailored messaging (for example, for Alaska Native, American Indian, Hispanic and migrant farmworker, African American, and disability communities), organized around the core message of consulting “someone you trust”. The federal role was therefore largely invisible to end recipients, who were reached through intermediaries whose own credibility did not depend on national institutional trust. We generalize this as a distinct strategy, “trust by proxy”, whereby institutions with low direct credibility can still support vaccine uptake by resourcing locally trusted messengers rather than attempting to rebuild their own credibility directly.

Recent studies support this interpretation. Exposure to misinformation alone does not uniformly lead to vaccine refusal; its effects appear to be mediated by trust in institutions and scientific authorities [28,45,46]. Individuals with high institutional trust are more likely to reject misinformation when they encounter it, whereas those with low institutional trust are more susceptible to its influence, suggesting that misinformation operates not by introducing entirely new beliefs but by reinforcing and legitimizing pre-existing skepticism. Likewise, educational attainment does not consistently predict vaccine acceptance when institutional distrust is high [29,42]; highly educated individuals who distrust institutions may be just as likely to refuse vaccination as those with lower educational levels. These observations suggest that trust functions not merely as one determinant among many but as a central lens through which information is interpreted and health decisions are made. The same piece of scientific evidence may be accepted or rejected depending on the credibility attributed to its source.

Within this perspective, vaccine confidence can be viewed as the downstream expression of a broader ecosystem of trust encompassing scientific institutions, public health authorities, regulatory agencies, and governments. Confidence in a specific vaccine is, in many ways, a proxy for confidence in the system that produced it and the authorities that recommend it. When trust in this ecosystem is high, individuals are more likely to accept new vaccines even in the presence of residual uncertainty about their long-term effects. When trust is low, even extensively tested and well-documented vaccines may be met with skepticism. Consequently, vaccination decisions are shaped not only by perceptions of vaccine safety and effectiveness, but also by confidence in the institutions responsible for generating, evaluating, and communicating health information. This perspective helps explain why vaccine hesitancy has persisted in some populations despite the availability of extensive scientific evidence supporting vaccine safety and effectiveness, the issue was not a lack of information but rather a crisis of legitimacy regarding the institutions responsible for generating and communicating that information [17,20,25].

The implication is profound. If confidence in institutions declines, efforts aimed solely at correcting misinformation or increasing knowledge may have limited effectiveness. Information is rarely evaluated in isolation; it is filtered through perceptions regarding the credibility of the source delivering it [39,41,49]. A communication strategy that assumes individuals will accept factual information if it is presented clearly ignores the reality that facts are interpreted through the lens of trust. In a context of institutional distrust, even the most accurate and well-presented information may be dismissed as biased, self-serving, or unreliable. This may explain why the unprecedented volume of scientific communication during the pandemic, including extensive public education campaigns, fact sheets, and expert briefings, failed to persuade many hesitant individuals. The problem was not that they lacked access to information; it was that they lacked trust in the sources providing it.

Institutional hesitancy therefore represents a potentially useful framework for understanding the evolving nature of vaccine acceptance in the post-pandemic world. Rather than replacing existing models, it complements them by emphasizing the broader social and political context in which vaccination decisions occur. The classical frameworks remain valuable for understanding individual-level factors, but they need to be situated within a wider understanding of institutional dynamics, political polarization, and the erosion of social trust. The conceptual transition from traditional vaccine hesitancy to institutional hesitancy is not a rejection of earlier work but an expansion of it, recognizing that vaccination decisions are shaped by forces that extend far beyond individual psychology. This framework has important implications for communication strategies, intervention design, and public health policy, implications that will be explored in the sections that follow.

### 2.3. From Hesitancy to Refusal: A Spectrum, Not a Category

The institutional hesitancy framework should not be read as implying a single, homogeneous population of “hesitant” individuals. Vaccine attitudes are better understood as a spectrum ranging from ready acceptance, through passive ambivalence and active hesitancy, to hardened refusal, a category that, unlike hesitancy proper, is largely closed to persuasion regardless of the information or access provided [1]. This distinction matters for the institutional hesitancy framework because institutional distrust appears to operate differently across this spectrum. For individuals in the hesitancy range, distrust interacts with, and is often outweighed by, more tractable factors such as convenience, complacency, and the credibility of a specific local messenger. By contrast, among those who have moved into outright refusal, institutional distrust is typically fused with a broader anti-institutional identity that extends well beyond vaccination, for example, distrust of mainstream media, public education, and electoral institutions, and functions less as a health attitude than as a marker of political and social identity. For this group, communication interventions are unlikely to shift behavior.

Online media environments plausibly contribute to this hardening. Algorithmic content curation and homophilous social networks create echo chambers in which individuals are repeatedly exposed to concordant anti-institutional narratives while encountering little corrective or countervailing information [12,13,52,53]. Repeated exposure within such closed networks does not merely fail to correct distrust; it can actively deepen it, providing continuous social reinforcement and a sense of shared identity around rejection of official guidance. Over time, this process may convert what began as situational hesitancy into a stable, identity-linked refusal that is resistant to subsequent correction, even when the individual’s objective access to accurate information improves.

Institutional distrust itself is not exclusively, or even primarily, a product of negative personal experience with institutions. A distinct and largely separate pathway runs through ideology: populist and anti-establishment political movements frame scientific, governmental, and media institutions as inherently self-serving, captured by hidden interests, or illegitimate, independent of those institutions’ actual conduct [15,19]. This ideological framing predates the COVID-19 pandemic and is not merely a reaction to it; the pandemic instead provided a high-salience occasion on which pre-existing ideological distrust could be mobilized and applied to a new domain. A defining feature of this pathway is that it tends to generalize across otherwise unrelated policy domains, for example, correlating institutional vaccine distrust with preference for home schooling driven by distrust of state education, or with rejection of official climate or electoral-integrity findings, which is consistent with the framing of institutional hesitancy as a manifestation of a broader relational stance toward institutions rather than a vaccine-specific belief.

## 3. Evidence and Limitations of Traditional Approaches

### 3.1. Evidence Supporting the Institutional Hesitancy Framework

A growing body of empirical evidence supports the notion that trust-related variables are among the strongest predictors of vaccination behavior across diverse populations and settings. This evidence, accumulated through large-scale surveys, cross-national comparisons, longitudinal studies, and meta-analyses, consistently demonstrates that institutional trust, rather than vaccine-specific concerns alone, plays a central role in shaping vaccine acceptance. The findings reviewed in this section provide robust support for the institutional hesitancy framework proposed in this paper, illustrating how trust in science, governments, public health authorities, healthcare systems, and regulatory bodies influences vaccination decisions across different contexts and populations.

Large population-based studies conducted in Europe, North America, and Asia have consistently demonstrated that trust in science is strongly associated with vaccine acceptance [18,26,27]. Individuals who perceive scientific institutions as credible, transparent, and motivated by public welfare are significantly more likely to view vaccines as safe, effective, and socially beneficial [26,37,44]. In a study conducted in Hungary, Kopasz and colleagues applied an extended Theory of Planned Behavior model and found that trust in science was a powerful predictor of COVID-19 vaccination intention, operating both directly and indirectly through perceived risk and knowledge [26]. Individuals with higher trust in science were more likely to perceive COVID-19 as a serious threat and to believe that vaccination was an effective protective measure. Similarly, Kara and colleagues demonstrated that trust in science was negatively associated with conspiracy beliefs and general vaccine hesitancy, with these variables mediating the relationship between trust and COVID-19 vaccine attitudes [27]. These findings suggest that trust in science functions as a foundational attitude that shapes how individuals evaluate vaccine-related information and make vaccination decisions. Individuals with high trust in science are more likely to accept scientific consensus on vaccine safety and effectiveness, while those with low trust are more susceptible to alternative narratives and conspiratorial thinking.

Similarly, trust in governments and public health authorities has repeatedly emerged as a major determinant of vaccine uptake [19,29,42]. In Austria, for example, confidence in governmental institutions was among the strongest predictors of vaccination status, surpassing traditional sociodemographic variables such as age, education, and income [29]. Individuals who expressed distrust in their government were significantly less likely to have received COVID-19 vaccination, even after controlling for other relevant factors. This pattern was not confined to Austria; comparable findings have been reported across multiple countries and cultural settings [18,19,54]. In Canada, Rizvi and colleagues documented that trust in federal and provincial governments, public health authorities, and health scientists was a consistent predictor of vaccine acceptance, with declines in trust over the course of the pandemic associated with lower vaccination rates [19]. These findings underscore the importance of political trust as a determinant of health behavior, particularly in the context of public health interventions that require collective compliance and sacrifice.

Cross-national investigations further highlight the importance of institutional factors. Chen and colleagues, in a cross-national survey, demonstrated that institutional trust significantly influenced vaccine attitudes across different countries, even after accounting for demographic and behavioral variables [28]. The study found that individuals who expressed high trust in their national governments and public health agencies were more likely to accept COVID-19 vaccines, while those who perceived these institutions as untrustworthy or self-interested were more hesitant. Bergmann and colleagues similarly found that national context plays a substantial role in shaping vaccine hesitancy across Europe, with variation in institutional trust, political polarization, and historical experiences explaining differences in vaccine acceptance between countries [54]. These findings suggest that broader institutional environments influence individual decision-making in ways that extend beyond personal attitudes, knowledge, or risk perception.

Evidence from low- and middle-income countries (LMICs), while more limited in the literature we identified, suggests both convergence and divergence from high-income patterns. As in the Nigerian data reported by Asaga and colleagues [47], institutional distrust and conspiracy endorsement predict vaccine refusal in LMIC settings as well, but this operates alongside, rather than in place of, dominant convenience and access barriers that remain comparatively more decisive determinants of uptake than in high-income countries. Baseline institutional credibility in many LMIC settings is also shaped by histories independent of COVID-19, including prior controversies around externally sponsored vaccine trials and colonial-era medical mistrust, meaning that institutional hesitancy in these contexts may be less a pandemic-era phenomenon than a pre-existing condition that the pandemic reactivated. The ’trust by proxy’ strategy gains particular relevance in these settings, given that community and religious institutions often hold comparatively greater relative authority than formal state institutions in shaping vaccine attitudes.

These observations underscore a broader principle: the institutional context in which vaccination decisions are made, including the perceived competence, transparency, and fairness of public institutions, shapes how individuals interpret health recommendations and make vaccination choices.

Evidence from minority and historically marginalized populations provides additional support for this framework. Studies among Black Americans have shown that trust in healthcare institutions and scientific authorities is closely linked to vaccine acceptance, often outweighing traditional demographic predictors such as age, education, or income [30,40]. Reinhart and colleagues found that trust in COVID-19 information from government and scientific sources was a strong predictor of vaccine acceptance among both Black and White Americans, though levels of trust differed significantly between groups [30]. Historical experiences of medical mistreatment, discrimination, and institutional racism have shaped perceptions of healthcare institutions in ways that influence vaccine attitudes, suggesting that vaccine decisions may reflect broader experiences with institutions rather than isolated evaluations of vaccine characteristics [40]. These findings indicate that for historically marginalized populations, vaccine hesitancy is often a manifestation of deeper institutional distrust rooted in lived experiences of exclusion, discrimination, and unequal treatment. Addressing this form of hesitancy requires not only improved communication about vaccines but also sustained efforts to address structural inequities and rebuild trust through meaningful community engagement.

Longitudinal evidence also points toward the central role of trust over time. The multinational study conducted by Lazarus and colleagues across 23 countries demonstrated that changes in confidence toward health information sources and public institutions influenced perceptions of routine immunization and preparedness for future health emergencies [18]. The study found that trust in healthcare professionals, scientists, and public health authorities had become more variable and fragile by 2023 compared to pre-pandemic baselines, with significant implications for vaccine confidence and public health resilience. Likewise, Rizvi and colleagues documented measurable shifts in trust toward health authorities, governments, and scientific experts during and after the pandemic, with these shifts correlating with changes in vaccination attitudes [19]. The erosion of trust observed during the pandemic has not been fully reversed, suggesting that the legacy of COVID-19 may persist for years and affect future vaccination programs, including routine immunization and pandemic preparedness efforts. These longitudinal data underscore that trust is not static but evolves over time in response to institutional behavior, communication, and broader social and political events.

Perhaps most importantly, several studies have shown that misinformation exerts its greatest influence when trust is already compromised [10,28,45,46,47,48]. The misinformation exposure-trust framework proposed by Zeng and colleagues suggests that misinformation effects are moderated by institutional trust; individuals with low trust in institutions are more susceptible to misinformation, while those with high trust are more likely to reject false claims [45]. Similarly, Asaga and colleagues found that conspiracy theory endorsement was associated with vaccine refusal primarily among individuals who already harbored distrust toward government and health institutions [47]. Duplaga and colleagues demonstrated that susceptibility to health misinformation was closely linked to general vaccine hesitancy, with trust in scientific institutions serving as a protective factor [46]. In this sense, misinformation may be better understood not as the root cause of vaccine hesitancy but as a force that exploits and amplifies existing vulnerabilities in institutional trust. Misinformation operates less by introducing entirely new beliefs and more by reinforcing pre-existing skepticism and providing narrative coherence to institutional distrust, offering seemingly coherent explanations for why institutions should not be trusted.

To provide a structured overview of the empirical foundation for the institutional hesitancy framework, Table 2 synthesizes the key studies by population, key finding, and implication. Across diverse geographic and demographic contexts, institutional trust, e.g., in science, governments, public health agencies, healthcare systems, and regulatory authorities, consistently predicts vaccine acceptance. The synthesis also reveals three recurring patterns: trust is dynamic and vulnerable to erosion during crises, it holds particular salience in historically marginalized populations, and it mediates how individuals process and respond to misinformation.

### 3.2. Limitations of Information-Based Approaches

For decades, public health responses to vaccine hesitancy have largely relied on a deficit model, assuming that individuals who refuse or delay vaccination do so primarily because they lack accurate information [39,55]. According to this perspective, improving knowledge should naturally increase vaccine acceptance: if people understand the science behind vaccination, appreciate the risks of vaccine-preventable diseases, and recognize the benefits of immunization, they will make the rational choice to vaccinate. This assumption, rooted in a broader tradition of science communication that emphasizes the transmission of factual knowledge from experts to lay audiences, has guided public health practice for generations. While intuitively appealing and sometimes effective in contexts where knowledge deficits are the primary barrier, the COVID-19 pandemic starkly demonstrated the limitations of this approach when trust is compromised [39,55]. The deficit model, it became clear, fails to account for the complex social, emotional, and institutional factors that shape how individuals interpret health information and make vaccination decisions.

One of the most striking observations of the pandemic era was that vaccine hesitancy frequently persisted despite unprecedented access to scientific information. Governments, public health agencies, scientific societies, healthcare institutions, and media organizations produced an enormous volume of educational material regarding COVID-19 vaccines, including detailed explanations of mRNA technology, safety data, clinical trial results, and public health recommendations [31,56,57,58]. Never before had so much scientific information been so readily available to the general public. Nevertheless, substantial segments of the population remained hesitant or resistant to vaccination, even in countries with high levels of education, widespread internet access, and intensive public health messaging. This persistence of hesitancy despite information abundance suggests that the problem is not simply one of knowledge gaps but rather one of trust gaps, a failure of credibility rather than a failure of communication.

This apparent paradox suggests that information alone is often insufficient to change attitudes when trust is compromised. Individuals rarely evaluate evidence in a purely objective manner, as the rational actor model of decision-making would suggest. Instead, information is interpreted through pre-existing beliefs, social identities, emotional experiences, and perceptions regarding the credibility of the source providing that information [14,41,49,59]. The same scientific data that is persuasive when delivered by a trusted physician may be dismissed as biased or self-serving when delivered by a government official or pharmaceutical company. This phenomenon, known as motivated reasoning, describes the tendency of individuals to process information in ways that align with their pre-existing beliefs and identities. When institutional trust is low, even the most carefully crafted educational materials may be interpreted through a lens of suspicion, with evidence being selectively scrutinized or dismissed entirely.

The rise of social media further complicates this landscape. Digital platforms have democratized access to information but have simultaneously weakened traditional gatekeeping mechanisms that once helped maintain some degree of quality control over public discourse [10,11,60,61]. In the pre-social media era, information was largely filtered through professional journalists, editors, and institutional experts who could verify claims and contextualize evidence. Today, anyone can publish content that reaches millions, often without editorial oversight or fact-checking. As a result, scientific evidence now competes directly with personal anecdotes, conspiracy theories, political narratives, and emotionally charged content for public attention [9,12,62]. The algorithmic amplification of engaging content, which often favors emotional, controversial, or sensational material over dry, factual information, further tilts the playing field against evidence-based communication. This creates an environment in which misinformation can spread faster and more widely than corrections, and where institutional voices struggle to compete with charismatic influencers and partisan commentators.

Importantly, misinformation does not operate solely by presenting false facts. Its effectiveness often derives from its ability to exploit existing distrust and reinforce perceptions that institutions are unreliable, self-interested, or deceptive [12,47,48]. Conspiracy theories, for example, often provide a coherent narrative that explains why institutional advice should be rejected: they present institutions as corrupt, captured by commercial interests, or engaged in hidden agendas. In this context, attempts to simply provide more information may have limited impact if the audience questions the legitimacy of the institutions delivering that information. More information from a source that is already viewed as untrustworthy is unlikely to change attitudes; it may even reinforce them by providing additional material for critical scrutiny. This dynamic, known as the backfire effect, occurs when corrections to misinformation inadvertently strengthen individuals’ commitment to their original beliefs.

This challenge has important implications for public health communication. Several recent studies suggest that successful communication strategies increasingly depend on empathy, transparency, dialogue, and community engagement rather than one-way information delivery [51,52,53,62]. Communication efforts that acknowledge uncertainty, address concerns respectfully, and involve trusted local actors appear to be more effective than approaches based solely on fact correction [49,50,51]. Empathetic communication that validates individuals’ concerns while providing context and evidence can build bridges of trust that make information more acceptable. Transparency about what is known, what remains uncertain, and how decisions are made can help demystify institutional processes and reduce perceptions of hidden agendas. Dialogue and community engagement, rather than top–down messaging, allow for the co-creation of communication strategies that are culturally appropriate and responsive to local concerns. Involving trusted local actors, such as community leaders, religious figures, and frontline healthcare workers, can leverage existing social trust to enhance the credibility of health messages.

The COVID-19 experience therefore suggests that rebuilding trust may be a prerequisite for restoring confidence in vaccines. Without trust, even accurate information may fail to achieve its intended effect. This insight has profound implications for public health practice. It suggests that investments in communication should not be limited to the production of educational materials but should extend to building and maintaining trust through consistent, transparent, and empathetic institutional behavior. It also suggests that public health authorities must engage with communities not as passive recipients of information but as active partners in health communication, respecting their concerns, addressing their needs, and involving them in decision-making processes.

**Figure 3:**
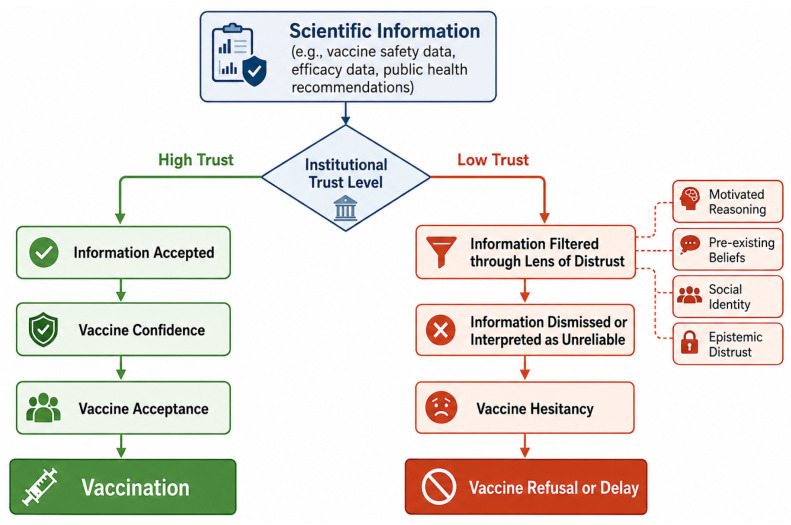
Flowchart illustrating why information alone is insufficient to change vaccine attitudes when institutional trust is compromised. The same scientific information, including vaccine safety data, efficacy data, and public health recommendations, leads to different outcomes depending on the level of institutional trust. In contexts of high trust (left path), information is accepted and translates directly into vaccine confidence and acceptance. In contexts of low trust (right path), the same information is filtered through motivated reasoning, pre-existing beliefs, social identity, and epistemic distrust, leading to vaccine hesitancy or refusal. This schematic highlights the fundamental limitation of traditional information-based approaches: without trust, information alone cannot overcome vaccine hesitancy.

The divergence in outcomes, where the same information leads to acceptance or refusal depending on the level of institutional trust, is illustrated in Figure 3. The figure shows two pathways: in contexts of high institutional trust (left path), scientific information is evaluated objectively, accepted, and translated into vaccine confidence and acceptance. In contexts of low institutional trust (right path), the same information is filtered through a lens of distrust, interpreted through motivated reasoning, pre-existing beliefs, social identity, and epistemic distrust, leading to vaccine hesitancy or refusal. This schematic highlights the fundamental limitation of information-based approaches: without trust, information alone cannot overcome vaccine hesitancy.

## 4. Rebuilding Trust in the Post-Pandemic Era: Implications and Strategies for Restoring Trust

The emergence of institutional hesitancy has implications that extend far beyond COVID-19 vaccination. If vaccine attitudes increasingly reflect broader perceptions of institutional trust, as the evidence reviewed in previous sections suggests, then future immunization programs may face challenges that cannot be addressed through traditional communication strategies alone. The pandemic has fundamentally altered the landscape of vaccine confidence, creating a legacy of institutional skepticism that may persist for years or even decades. This shift has profound implications for routine immunization programs, seasonal vaccination campaigns, efforts to introduce new vaccines, and preparedness for future pandemics. Understanding these implications is essential for designing effective, resilient vaccination strategies in the post-pandemic era.

This issue is particularly relevant because the effects of the pandemic are already influencing attitudes toward routine immunization. Evidence from several countries indicates that confidence in vaccination programs has become more volatile since COVID-19, affecting perceptions of vaccines beyond those specifically developed for SARS-CoV-2 [18,38,56]. The dynamic and fragile nature of vaccine confidence, which Larson and Broniatowski described as susceptible to rapid fluctuations in response to social and political events, has been starkly illustrated during the pandemic [38]. The heightened public scrutiny, political polarization, and intense media coverage that characterized COVID-19 vaccination have created an environment in which vaccine confidence is more easily disrupted. This volatility has not been confined to COVID-19 vaccines; it has spilled over into attitudes toward routine childhood immunizations, adult vaccines, and other preventive health measures. Vojtek and colleagues documented that the pandemic has had a measurable impact on vaccine confidence and uptake across multiple vaccine types, with significant implications for public health programs worldwide [56].

Future pandemic preparedness may be particularly affected by institutional hesitancy. During future outbreaks, public compliance with vaccination campaigns, non-pharmaceutical interventions, and risk communication strategies will depend heavily on the level of trust established before a crisis occurs [18]. Trust in institutions is built over long periods through consistent, transparent, and accountable behavior. Once institutional trust has been eroded, rebuilding it during an emergency may prove exceptionally difficult, as the urgency of the situation often demands rapid action that may not allow for the careful trust-building processes that are normally required. The COVID-19 experience demonstrated that trust is essential for both vaccine acceptance and adherence to public health measures such as masking, physical distancing, and testing. A population that has lost trust in its institutions is less likely to comply with public health recommendations, potentially prolonging outbreaks and increasing morbidity and mortality. Lazarus and colleagues found that post-pandemic trust patterns influenced perceptions of preparedness for future health emergencies, suggesting that the legacy of COVID-19 may affect pandemic response for years to come [18]. This finding underscores the urgency of rebuilding trust as a foundational element of pandemic preparedness.

These effects are already visible for specific vaccine programs. Seasonal influenza vaccination, which requires annual re-engagement and active scheduling, is particularly vulnerable to erosion in institutional confidence, and disproportionately affects older adults, young children, and people with chronic conditions who already experience coverage disparities [20]. Human papillomavirus (HPV) vaccination, historically subject to its own controversies around adolescent sexuality and safety, may face compounding barriers where parents who became skeptical of public health authorities during the pandemic extend that skepticism to adolescent immunization more broadly [43]. The resurgence of measles in several countries similarly reflects, alongside disrupted routine services and misinformation exposure, an underlying erosion of institutional trust required to sustain the very high (approximately 95%) coverage needed for herd immunity [43]. Encouragingly, frontline vaccine confidence practitioners report anecdotally that active local measles outbreaks are prompting a resurgence of catch-up MMR vaccination among previously hesitant parents, consistent with the classical complacency mechanism (perceived risk rising once disease becomes locally visible).

The concept of institutional hesitancy also has implications for emerging vaccine technologies. Acceptance of novel platforms, including next-generation mRNA vaccines and other innovative approaches, is likely to depend not only on scientific evidence but also on confidence in the institutions responsible for their development and regulation [22]. The unprecedented speed of mRNA vaccine development during the pandemic generated both admiration and suspicion. While some individuals viewed this as a triumph of science, others questioned the safety of vaccines developed so rapidly, expressing concerns about regulatory shortcuts and commercial pressures. These concerns may carry over to new vaccines developed with similar technologies, even when extensive safety data are available. Wong and colleagues found that confidence in regulatory authorities and vaccine manufacturers was a significant predictor of acceptance of locally manufactured mRNA vaccines, suggesting that institutional trust will play a critical role in the uptake of future vaccine technologies [22]. This has implications for the development and deployment of vaccines against other infectious diseases, as well as for vaccines targeting non-infectious conditions such as cancer.

These considerations suggest that vaccine programs should increasingly be viewed as components of broader trust ecosystems. Maintaining high vaccination coverage may require sustained investment in institutional credibility, transparency, and public engagement rather than relying exclusively on vaccine-specific interventions. This shift in perspective has significant implications for public health policy and practice. It suggests that investments in vaccine confidence should not be limited to communication campaigns about specific vaccines but should extend to building and maintaining trust in the institutions that develop, regulate, recommend, and deliver vaccines. It also suggests that public health authorities must engage with communities in sustained partnerships, addressing the structural and historical factors that have eroded trust, particularly among marginalized populations. As the institutional hesitancy framework proposed in this review suggests, the challenge facing vaccination programs is not merely a communication challenge; it is a trust challenge that requires sustained institutional commitment.

These strategies should not be read as uniformly applicable. Consistent with the hesitancy–refusal distinction introduced in Section 2.3, community engagement, transparency, and trusted local messengers are likely to be effective chiefly for populations whose reluctance remains within the hesitancy range, including many historically marginalized communities whose distrust is grounded in specific, addressable institutional conduct and lived experience. For individuals who have moved into ideologically hardened refusal, by contrast, our recommendations are unlikely to be sufficient, since their distrust is not primarily a response to the conduct of health institutions but an expression of a broader political identity extending to unrelated institutions. Shifting this population’s attitudes, to the extent it is achievable at all through public health means, would require system-wide changes in political discourse, media accountability, and social cohesion that lie substantially outside the remit of vaccination programs. We therefore suggest that realistic public health objectives for this subgroup should shift from persuasion toward harm reduction, for example, protecting immunocompromised and other vulnerable contacts, and maintaining herd immunity thresholds through high coverage in the remainder of the population, rather than assuming universal convertibility to vaccine acceptance.

If declining institutional trust represents a major driver of vaccine hesitancy, as the evidence reviewed in previous sections suggests, then restoring confidence becomes one of the most important challenges facing public health in the post-pandemic era. The erosion of trust documented during the COVID-19 pandemic has created a legacy of institutional skepticism that may persist for years, affecting not only COVID-19 vaccination but also routine immunization programs, seasonal vaccination campaigns, and preparedness for future health emergencies. Addressing this challenge requires a fundamental shift in how public health institutions approach their relationship with citizens, moving beyond communication campaigns and educational interventions toward sustained, transparent, and accountable institutional behavior.

Trust cannot be mandated, nor can it be restored through communication campaigns alone. Unlike compliance, which can be enforced through regulations or mandates, trust is a relational construct that emerges through repeated interactions in which institutions demonstrate competence, transparency, consistency, fairness, and accountability [17,21,37]. Trust is built over time through a track record of reliable behavior, honest communication, and genuine responsiveness to public concerns. It cannot be demanded or manufactured through public relations efforts; it must be earned through consistent institutional conduct. This understanding has profound implications for public health practice, suggesting that investments in trust-building should be viewed as long-term commitments rather than short-term interventions.

Transparency is likely to be particularly important in rebuilding trust. During the COVID-19 pandemic, uncertainty was often perceived negatively because it was interpreted as weakness, inconsistency, or even dishonesty. However, evidence suggests that openly communicating uncertainty may actually strengthen credibility when performed honestly and consistently [14,17,41]. Public trust is more likely to be preserved when institutions explain what is known, what remains uncertain, and how recommendations may evolve as new evidence emerges. Rather than presenting scientific knowledge as static and certain, institutions can build trust by acknowledging the provisional nature of scientific understanding while providing clear guidance based on the best available evidence. This approach, sometimes described as honest brokerage, recognizes that transparency about uncertainty can enhance credibility by demonstrating that institutions are not hiding inconvenient truths or overstating their confidence. During future health emergencies, public health authorities should therefore prioritize transparent communication about the limits of current knowledge, the rationale for evolving recommendations, and the processes by which decisions are made.

Community engagement represents another essential strategy for rebuilding trust. Several studies have highlighted the effectiveness of approaches that actively involve communities in health communication rather than treating them as passive recipients of information [51,52]. Partnerships with local leaders, healthcare professionals, community organizations, and trusted social networks may be particularly valuable in populations characterized by historical distrust [30,40,52]. Community engagement recognizes that trust is not solely a function of institutional behavior but also of social relationships and networks. When communities are actively involved in the design and implementation of health programs, they are more likely to view those programs as legitimate and responsive to their needs. This is particularly important for historically marginalized populations, who may have experienced discrimination, neglect, or exploitation by healthcare institutions. For these communities, trust cannot be restored through generic communication campaigns; it requires sustained engagement, meaningful partnership, and demonstrated commitment to addressing the structural inequities that have eroded trust.

Healthcare workers continue to play a central role in trust-building efforts. Surveys consistently show that physicians, nurses, and other frontline healthcare professionals remain among the most trusted sources of health information, often ranking above government officials, public health agencies, and media outlets [41,43]. The trust that individuals place in their healthcare providers is rooted in personal relationships, face-to-face interactions, and perceptions of care and concern. Supporting healthcare workers with effective communication training may therefore strengthen vaccine confidence at the community level [50]. Training programs that equip healthcare workers with skills in empathetic communication, motivational interviewing, and addressing vaccine concerns can enhance their ability to build trust with patients. Additionally, supporting healthcare workers themselves, through adequate staffing, fair compensation, and protection from burnout, is essential for maintaining the trust that patients place in them. Healthcare workers who are overburdened and stressed are less able to provide the empathetic, patient-centered care that builds trust.

Public health messaging may also benefit from a shift in tone. Messages focused exclusively on correcting misinformation can sometimes reinforce polarization and defensive reactions, particularly when individuals feel that their concerns are being dismissed or that they are being lectured. Alternative approaches based on empathy, dialogue, storytelling, and shared values have shown promising results in promoting vaccine confidence [49,51,53]. Empathetic communication that validates individuals’ concerns while providing context and evidence can build bridges of trust that make information more acceptable. Storytelling that connects vaccination to personal experiences, community values, and shared goals can be more persuasive than abstract data or impersonal statistics. Dialogue and conversation, rather than one-way messaging, allow for the co-creation of meaning and the development of mutual understanding. These approaches recognize that health communication is not merely about transmitting information but about building relationships and fostering shared commitment to public health goals.

Finally, rebuilding trust requires addressing the broader social and political conditions that contribute to institutional skepticism. Polarization, inequality, exclusion, and perceived lack of accountability can undermine confidence in public institutions regardless of the quality of scientific evidence available [13,16,17]. When societies are deeply divided along political, racial, or socioeconomic lines, trust in institutions tends to fracture along those same lines. Individuals who feel excluded from political processes, marginalized by economic systems, or discriminated against by social institutions are less likely to trust those institutions, regardless of their performance. Consequently, efforts to improve vaccine acceptance cannot be separated entirely from wider discussions regarding governance, social cohesion, and public engagement. Addressing the root causes of institutional distrust, including political polarization, social inequality, and historical injustices, may be essential for building the broad-based trust that vaccination programs require.

To provide a practical framework for translating these insights into action, Table 3 synthesizes the five core strategies discussed above: transparency, community engagement, healthcare worker support, empathetic messaging, and addressing broader social and political conditions. For each strategy, the table outlines concrete actions that institutions can implement, expected outcomes, and supporting evidence from the literature. The synthesis reveals that rebuilding trust is not a single intervention but a multi-dimensional effort requiring sustained commitment across multiple fronts. Several patterns emerge: transparency as a foundation for credibility, the critical role of healthcare workers as trusted intermediaries, and the recognition that trust-building cannot be separated from addressing the structural inequities that have historically eroded institutional credibility.

Rebuilding trust is therefore not simply a communication challenge. It is an institutional challenge that requires sustained commitment to transparency, community engagement, support for healthcare workers, and addressing the broader social and political conditions that shape public confidence. The pandemic has exposed deep fractures in the relationship between citizens and institutions, and repairing these fractures will require more than better messaging or improved access to vaccination services. It will require fundamental changes in how public health institutions operate, communicate, and engage with the communities they serve. This challenge is substantial, but it is also essential for restoring vaccine confidence and ensuring the resilience of public health systems in the face of future health threats.

## 5. Discussion

The COVID-19 pandemic has fundamentally altered the landscape of vaccine acceptance, exposing deep fractures in the relationship between citizens and institutions that previous frameworks failed to fully capture. Our review synthesizes evidence from multiple countries, populations, and methodological approaches to demonstrate that institutional trust has emerged as a central determinant of vaccination behavior, often surpassing traditional predictors such as education, risk perception, or knowledge about vaccines. This finding challenges the deficit model that has long dominated public health thinking and calls for a fundamental reconceptualization of vaccine hesitancy.

The institutional hesitancy framework we propose offers several theoretical and practical contributions to the field. First, it shifts the analytical focus from individual-level psychological constructs to relational dynamics between citizens and institutions. This is not merely a semantic distinction; it has profound implications for how we understand, measure, and address vaccine reluctance. Traditional frameworks such as the “3C” and “5C” models remain valuable for assessing individual determinants, but they are insufficient to explain why similar information is interpreted differently across populations with varying levels of institutional trust. By placing trust at the center of the analytical framework, we acknowledge that vaccination decisions are embedded within broader social, political, and historical contexts that shape how individuals interpret health recommendations. Second, the institutional hesitancy framework helps explain why misinformation has proven so difficult to counteract through traditional fact-checking approaches. Our review indicates that misinformation operates less by introducing entirely new beliefs and more by reinforcing and legitimizing pre-existing institutional distrust. Individuals who already harbor skepticism toward governments, public health agencies, or scientific institutions are more susceptible to conspiratorial narratives that confirm their suspicions. This suggests that efforts to combat misinformation cannot be separated from efforts to rebuild institutional trust. Fact-checking campaigns that do not address the underlying trust deficit may have limited impact, as they fail to address the relational dynamics that make misinformation persuasive in the first place. Third, our framework has important implications for the design of public health communication strategies. The evidence reviewed here suggests that effective communication in the post-pandemic era requires a shift from one-way information delivery to dialogue, transparency, and community engagement. Communication strategies that acknowledge uncertainty, address concerns empathetically, and involve trusted local actors appear more effective than approaches based solely on fact correction. This shift requires public health institutions to move beyond the assumption that knowledge deficits drive hesitancy and to recognize that trust must be earned through consistent, transparent, and accountable institutional behavior. The COVID-19 experience has demonstrated that trust is not a static attribute but a dynamic relational construct that can be eroded or rebuilt through institutional actions.

Several limitations of this review should be acknowledged. First, as a narrative review, our synthesis is subject to selection bias and the interpretive judgments of the authors. While we have drawn on a wide range of evidence from multiple countries and settings, the review is not a systematic meta-analysis and does not provide quantitative estimates of the strength of associations between trust and vaccine acceptance. Second, the evidence base remains geographically concentrated, with most studies originating from Western Europe, North America, and a limited number of other high-income settings. Data from low- and middle-income countries, as well as from Eastern and Southern Europe, are comparatively limited, constraining the generalizability of our findings. Third, the concept of institutional hesitancy requires further empirical validation, including the development and testing of measurement instruments that can capture its multidimensional nature across different cultural and institutional contexts. Fourth, our review focuses primarily on trust in health-related institutions; we do not fully explore the role of other forms of institutional trust, such as trust in media, educational systems, or the broader political system, which may also shape vaccine attitudes. A further limitation concerns causal inference: because the evidence synthesized here is overwhelmingly cross-sectional and observational, the associations we describe between institutional trust and vaccine acceptance cannot establish directionality, and residual confounding, most notably by political ideology, which may independently shape both institutional distrust and vaccine refusal, cannot be excluded. We have accordingly framed institutional hesitancy throughout as a descriptive and organizing model rather than a causal one, and we use associational rather than causal language when summarizing individual studies. In addition, evidence from low- and middle-income countries remains comparatively sparse in the literature we were able to identify; where convenience and access barriers remain dominant, and where community or religious institutions may carry more relative weight than formal state institutions, the dynamics of institutional hesitancy may differ substantially from the largely high-income evidence base reviewed here, and this constrains the generalizability of our conclusions.

Despite these limitations, the evidence consistently points to the central role of institutional trust in shaping vaccination behavior. This finding has significant implications for public health policy, clinical practice, and future research. This reframing parallels an earlier shift in the health literacy field, from an individual-deficit model, in which limited health literacy was treated as a fixed attribute of the person, toward a systems- or institutional-responsibility model, in which the demands and clarity of the health system itself are treated as the primary lever for improving outcomes. We propose that vaccine confidence is amenable to the same conceptual move: rather than treating hesitancy solely as a property of individuals to be corrected, institutional hesitancy relocates a substantial share of the responsibility, and therefore the locus of intervention, to the institutions themselves. Several avenues for future research emerge from our analysis. First, longitudinal studies are needed to track changes in institutional trust and their effects on vaccine acceptance over time, particularly as the legacy of the pandemic continues to unfold. The dynamic nature of trust, which can fluctuate in response to institutional actions, political events, and media coverage, requires repeated measurement to capture its evolving influence. Second, comparative research across countries with different institutional contexts, political systems, and historical experiences of trust erosion would help identify contextual factors that moderate the relationship between trust and vaccine acceptance. Third, intervention studies that test strategies for rebuilding trust, such as community engagement programs, transparency initiatives, and institutional reforms, would provide valuable evidence for public health practice. Fourth, research on the relationship between institutional trust and vaccine acceptance in historically marginalized populations is urgently needed, as these communities often bear the greatest burden of institutional distrust and vaccine-preventable diseases. In addition, future research should explore the mechanisms through which institutional trust influences vaccine behavior. Our review suggests multiple pathways, including direct effects on risk perception, indirect effects through susceptibility to misinformation, and moderating effects on the interpretation of health recommendations. Understanding these mechanisms would enable more targeted and effective interventions. Finally, the institutional hesitancy framework should be validated across different vaccine types, including routine childhood immunizations, seasonal vaccines, and novel vaccine technologies, to assess its generalizability beyond the COVID-19 context.

## 6. Conclusions

The COVID-19 pandemic has left an enduring legacy that extends far beyond the direct health impacts of the virus. It has fundamentally reconfigured the relationship between citizens, science, and public institutions, creating a landscape in which vaccine acceptance is increasingly shaped by perceptions of institutional credibility, transparency, and legitimacy. The institutional hesitancy framework proposed in this review offers a new lens for understanding this transformation, shifting the analytical focus from individual knowledge deficits to the relational dynamics between institutions and the communities they serve.

Several key messages emerge from our analysis. First, institutional trust is not merely one determinant among many but a central lens through which all other determinants of vaccine behavior are filtered. Efforts to address vaccine hesitancy that neglect the trust deficit are unlikely to succeed, regardless of their scientific accuracy or communication sophistication. Second, the erosion of trust observed during the pandemic has created a legacy of institutional skepticism that extends beyond COVID-19 vaccines to routine immunization programs, seasonal vaccination campaigns, and future pandemic preparedness. Rebuilding trust will require sustained effort, transparency, accountability, and community engagement over years or decades, not months. Third, healthcare professionals remain among the most trusted sources of health information; supporting them with effective communication skills and protecting them from burnout is essential for maintaining vaccine confidence at the community level.

The implications for public health practice are clear. Vaccination programs must be viewed not merely as technical interventions but as components of broader trust ecosystems. Investments in vaccine confidence should extend beyond communication campaigns to include institutional reforms, community partnerships, and sustained engagement with marginalized populations. Public health authorities must prioritize transparency about uncertainty, acknowledge past failures, and demonstrate genuine responsiveness to public concerns. This requires a fundamental shift in how institutions approach their relationship with citizens, moving from a top–down, information-deficit model to a collaborative, trust-centered approach.

Ultimately, the success of vaccination programs in the post-pandemic era will depend not only on the quality of vaccines or the effectiveness of delivery systems but also on the ability of public health institutions to rebuild trust with the communities they serve. The institutional hesitancy framework provides a conceptual foundation for this work, but its realization requires sustained commitment, political will, and genuine institutional transformation. As we prepare for future health emergencies, the lessons of COVID-19 must not be forgotten: trust is the invisible infrastructure of public health, and without it, even the most effective interventions will fail to achieve their potential. Restoring that trust is not merely a communication challenge but a fundamental obligation of public health institutions in the post-pandemic era.

## Figures and Tables

**Table 1:** Comparison of classical vaccine hesitancy and the institutional hesitancy framework across multiple conceptual dimensions, highlighting the shift from a vaccine-centered, individual-level perspective to a trust-centered, institution-level approach.

Domain	Classical Vaccine Hesitancy	Institutional Hesitancy
Primary object of concern	The vaccine itself (safety, effectiveness, necessity) [1,2,5,6,7,31,32]	The institutions responsible for developing, regulating, recommending, and communicating vaccines [17,18,19,20,21,22,23,24,25]
Analytical focus	Individual attitudes and decision-making processes [1,2,6,7,31]	Relationship between citizens and institutions [17,18,19,23,25]
Main determinants	Confidence, complacency, convenience, constraints, calculation, collective responsibility [3,5,6,7]	Trust in science, governments, public health agencies, healthcare systems, and regulatory authorities [18,19,22,26,27,28,29,30]
Role of trust	One determinant among several influencing vaccine acceptance [2,35,37]	Central framework through which information and recommendations are interpreted [17,18,19,26,27,28,29,30]
Role of misinformation	Directly influences vaccine attitudes and risk perceptions [9,10,11,12,31,32,33,34,35,36]	Amplifies pre-existing distrust toward institutions and experts [28,45,46,47,48]
Interpretation of scientific uncertainty	Usually considered a challenge for risk communication [14,39]	May be perceived as evidence of institutional inconsistency or lack of credibility [14,24,41]
Primary explanation for refusal or delay	Concerns about vaccine safety, efficacy, adverse events, or disease risk [31,32,33,34,35,36]	Skepticism regarding the legitimacy, transparency, or trustworthiness of institutions [17,20,23,25]
Communication model	Information provision and correction of misconceptions [3,6,39]	Dialogue, transparency, community engagement, and trust-building [49,50,51,52,53]
Public health interventions	Education campaigns, improved access, vaccine promotion [3,5,6,7,39]	Institutional transparency, stakeholder engagement, accountability, and long-term trust restoration [17,21,50,51,52,53]
Underlying question	“Do I trust this vaccine?”	“Do I trust the institutions behind this vaccine?”
Expected outcome of successful intervention	Increased vaccine confidence and uptake [3,5,6,7,31,32]	Restoration of institutional trust, leading to sustainable vaccine confidence and broader public health resilience [17,18,19,20,21,50,51,52,53]

**Table 2:** Empirical evidence for the institutional hesitancy framework, summarizing key studies that demonstrate the central role of institutional trust in vaccine acceptance across diverse populations and settings. *Note*: Consistency of evidence categories: “Strong and consistent” indicates that findings are robust across multiple studies and settings with minimal contradictory evidence; “Consistent but context-dependent” indicates that findings are generally reproducible but vary in magnitude or direction across populations or settings; “Weak/context-dependent” indicates limited or mixed evidence requiring further investigation.

Study	Population/Setting	Key Finding	Implication for Institutional Hesitancy	Consistency of Evidence
Larson et al. (2016) [2]	67 countries	Substantial global variability in vaccine confidence, strongly influenced by trust in health systems and scientific authorities	Vaccine confidence is shaped by institutional trust at a population level	Strong and consistent
Lazarus et al. (2024) [18]	23 countries	Confidence in health information sources and public institutions became more variable and fragile post-pandemic	Institutional trust is dynamic and susceptible to erosion during health crises	Strong and consistent
Rizvi et al. (2026) [19]	Canada	Measurable declines in trust toward governments and public health authorities during the pandemic, correlated with vaccination attitudes	Trust erosion has measurable effects on vaccine acceptance	Consistent but context-dependent
Kopasz et al. (2026) [26]	Hungary	Trust in science is a powerful predictor of COVID-19 vaccination intention, operating through perceived risk and knowledge	Trust in science functions as a foundational attitude shaping vaccine decisions	Strong and consistent
Kara et al. (2025) [27]	Turkey	Trust in science negatively associated with conspiracy beliefs and general vaccine hesitancy	Trust in science protects against misinformation and hesitancy	Consistent but context-dependent
Schernhammer et al. (2022) [29]	Austria	Confidence in governmental institutions was among the strongest predictors of vaccination status	Political trust is a critical determinant of vaccine acceptance	Strong and consistent
Chen et al. (2022) [28]	Cross-national	Institutional trust significantly influenced vaccine attitudes across different countries	Trust effects generalize across cultural and political contexts	Strong and consistent
Reinhart et al. (2022) [30]	USA (Black and White Americans)	Trust in COVID-19 information from government and scientific sources was a strong predictor of vaccine acceptance	Trust is particularly important in historically marginalized populations	Strong and consistent
Zeng et al. (2026) [45]	USA (college students)	Misinformation effects are moderated by institutional trust; high trust protects against misinformation	Trust functions as a filter through which information is interpreted	Strong and consistent
Duplaga et al. (2026) [46]	Poland	Susceptibility to health misinformation is closely linked to general vaccine hesitancy; trust in science is protective	Trust in science is a protective factor against misinformation effects	Consistent but context-dependent
Asaga et al. (2026) [47]	Nigeria	Conspiracy theory endorsement associated with vaccine refusal primarily among those with institutional distrust	Misinformation exploits and amplifies pre-existing institutional distrust	Consistent but context-dependent
Bergmann et al. (2026) [54]	Europe (multiple countries)	National context plays a substantial role in shaping vaccine hesitancy	Institutional environments influence individual decision-making	Consistent but context-dependent
Unspecified multi-country studies on access barriers in lower-resource and rural settings (e.g., convenience and structural constraints literature [6])	Lower-resource and rural settings	Institutional trust effects attenuate substantially once access/convenience barriers are entered into models, which sometimes dominate	Institutional trust is necessary but not sufficient where structural access barriers remain unresolved	Weak/context-dependent
High-trust ceiling-effect settings referenced in Larson et al. (2016) [2]	Countries with historically very high baseline institutional trust	Additional gains in institutional trust show diminishing marginal association with vaccine acceptance once trust is already high	The institutional hesitancy framework may have greatest explanatory value precisely where trust is contested or declining, rather than uniformly	Weak/context-dependent

**Table 3:** Strategies for rebuilding institutional trust in the post-pandemic era. The table summarizes six key strategies, i.e., transparency, community engagement, healthcare worker support, empathetic messaging, addressing broader social and political conditions, and trust by proxy, with concrete actions, expected outcomes, supporting references, and strength of evidence. These strategies provide a practical framework for public health institutions seeking to restore trust and strengthen vaccine confidence. *Note:* Strength of evidence categories: “Strong” indicates robust evidence from multiple controlled or survey studies with consistent findings; “Moderate” indicates consistent but limited evidence, often from observational or qualitative studies; “Emerging/indirect” indicates preliminary or indirect evidence (e.g., programmatic examples, case studies) requiring further controlled evaluation.

Strategy	Key Actions	Expected Outcome	Supporting References	Strength of Evidence
Transparency	Communicate uncertainty honestly; explain decision-making processes; acknowledge evolving evidence; share rationale for recommendations	Enhanced institutional credibility; reduced suspicion; increased public understanding of scientific processes	[14,17,41]	Moderate (mostly observational/qualitative)
Community Engagement	Partner with local leaders; involve communities in program design; co-create culturally appropriate communication strategies; engage trusted social networks	Greater program legitimacy; responsive to local needs; improved trust in historically marginalized populations	[30,40,51,52]	Strong (multiple controlled and survey studies, including marginalized-population evidence)
Healthcare Worker Support	Provide communication training (empathetic communication, motivational interviewing); ensure adequate staffing, fair compensation, and burnout prevention	Stronger patient trust; more effective vaccine conversations; sustained healthcare workforce	[41,43,50]	Strong
Empathetic Messaging	Use dialogue over lecture; incorporate storytelling; validate concerns; emphasize shared values and collective responsibility	Reduced polarization; increased vaccine confidence; greater message acceptance	[49,51,53]	Moderate
Address Social/Political Conditions	Tackle inequality and exclusion; promote social cohesion; ensure accountability; address historical injustices and structural barriers	Broad-based trust; sustainable public health resilience; reduced institutional skepticism	[13,16,17]	Emerging/indirect (no direct intervention studies to date)
Trust by Proxy	Fund and empower locally/culturally trusted intermediaries (community organizations, local providers, faith leaders) rather than communicating directly from distal institutions; tailor messaging by community while keeping the funding institution’s role largely invisible to recipients	Vaccine uptake among populations with low trust in distal institutions but higher trust in proximal messengers	National COVID-19 Resiliency Network example [30,40,51,52]	Emerging/indirect (programmatic case example; limited controlled evaluation)

## Data Availability

No new data were created or analyzed in this study. Data sharing is not applicable to this article.
